# Estimation of Bladder Pressure and Volume from the Neural Activity of Lumbosacral Dorsal Horn Using a Long-Short-Term-Memory-based Deep Neural Network

**DOI:** 10.1038/s41598-019-54144-8

**Published:** 2019-12-02

**Authors:** Milad Jabbari, Abbas Erfanian

**Affiliations:** Department of Biomedical Engineering, School of electrical engineering, Iran Neural Technology Research Center, Iran University of Science and Technology (IUST), Tehran, Iran

**Keywords:** Biomedical engineering, Mathematics and computing

## Abstract

In this paper, we propose a deep recurrent neural network (DRNN) for the estimation of bladder pressure and volume from neural activity recorded directly from spinal cord gray matter neurons. The model was based on the Long Short-Term Memory (LSTM) architecture, which has emerged as a general and effective model for capturing long-term temporal dependencies with good generalization performance. In this way, training the network with the data recorded from one rat could lead to estimating the bladder status of different rats. We combined modeling of spiking and local field potential (LFP) activity into a unified framework to estimate the pressure and volume of the bladder. Moreover, we investigated the effect of two-electrode recording on decoding performance. The results show that the two-electrode recordings significantly improve the decoding performance compared to single-electrode recordings. The proposed framework could estimate bladder pressure and volume with an average normalized root-mean-squared (NRMS) error of 14.9 ± 4.8% and 19.7 ± 4.7% and a correlation coefficient (CC) of 83.2 ± 3.2% and 74.2 ± 6.2%, respectively. This work represents a promising approach to the real-time estimation of bladder pressure/volume in the closed-loop control of bladder function using functional electrical stimulation.

## Introduction

The lower urinary tract is responsible for the accumulation of urine (continence) and elimination of urine (micturition) at an appropriate time. Neurological disease or spinal cord injury (SCI) can disrupt the normal functions of the lower urinary tract, resulting in detrusor sphincter dyssynergia (DSD) and neurogenic detrusor hyperreflexia (NDO)^1^. NDO is characterized by involuntary detrusor contractions during the filling phase even at small bladder volumes, while DSD consists of simultaneous contractions of the bladder and the urethral sphincter. DSD could lead to incomplete elimination of urine and high transient bladder pressures and NDO to low bladder capacity and incontinence.

Electrical stimulation has been used as an alternative approach to traditional methods for the treatment of neurogenic lower urinary tract dysfunction^2^. Many studies have demonstrated that reflex bladder inhibition can be achieved by electrical stimulation of the inhibitory pathways of pudendal nerve (PN) afferents to treat NDO^3–5^. Moreover, it has been shown that dorsal genital nerve stimulation can suppress undesired detrusor bladder contractions in patients with NDO^6^. It has also been demonstrated that stimulating and blocking of the pudendal nerves^7,8^, high frequency electrical stimulation of the pudendal nerve and sacral root stimulation^9^, and stimulating and high frequency blocking of the sacral nerve roots^10^ can eliminate external urethral sphincter activation and restore bladder voiding. To produce micturition with reduced bladder-sphincter dyssynergia, a combination of low frequency and high frequency stimulation of the pelvic nerve has been investigated^11^. The low frequency stimulation of the distal pelvic nerve was provided to evoke bladder contraction, and proximal high frequency stimulation of the pelvic nerve was used to block afferent activation.

Electrical stimulation to restore bladder functions can be applied continuously or conditionally^12–16^. Continuous stimulation may increase the risk of tissue damage due to the energy deployed during the stimulation and the risk of implanted electrode corrosion and may lead to habituation of the spinal reflexes. Moreover, continuous stimulation requires high power consumption with respect to conditional stimulation.

To overcome the limitations of continuous stimulation, conditional stimulation can be employed, where the stimulation is applied when an impending bladder contraction is set to occur or the bladder requires voiding. To inhibit impeding bladder contraction by conditional stimulation, it is necessary to detect the onset of nascent hyperreflexive contractions.

Several methods for detecting bladder contractions and triggering stimulation have been reported. One common approach is to monitor intravesical pressure using artificial sensors^17–23^. However, there are a number of challenges facing artificial sensors that need to be addressed before clinical trials can be considered. These challenges are primarily related to invasiveness, artifacts from patient movement, abdominal pressure changes^24^, material biocompatibility^25^, and related instruments’ decreasing reliability over time^26,27^. Several alternative approaches have also been proposed to measure either the intravesical pressure or volume, using electromyography (EMG) of the external urethral sphincter^6,28–30^, electroneurography (ENG) of the pudendal nerve trunk using cuff-electrodes^5,31^, pudendal nerve activity using penetrating intrafascicular electrodes^32^, and ENG of the pelvic nerve33 and sacral nerve roots^33,34^. However, several issues, such as a high degree of invasiveness, motion artifacts caused by organ movement, and low signal-to-noise ratios of electroneurograms, limit the chronic monitoring of intravesical pressure/volume. Moreover, ENG recordings from either the pudendal nerve^5,31^, sacral root nerve^33,34^ or pelvic nerves^33^ using cuff electrodes provide a whole-nerve action potential composite of several units in the nerve that also carries information from other sources.

Multi-channel recordings from dorsal root ganglia (DRG)^27,35^ or dissected intradural dorsal rootlets^36^ have also been considered as a potential alternative to pressure/volume estimation. The DRG is a cluster of sensory neurons that conveys information from the skin, muscles, and joints of the limbs and trunk to the spinal cord. Several studies have also demonstrated the feasibility of deriving limb-state estimates from the firing rates of primary afferent neurons recorded in DRG^37–45^. An important challenge facing DRG recordings is the long-term chronic recordings from DRGs. Recently, it has been demonstrated that the longest continuously tracked bladder afferent could last for 23 days^46^.

Recently, neural activity recorded from the dorsal horn of the spinal cord has been used to demonstrate the feasibility of estimating the pressure^26^ and volume^47^ of the bladder. For this purpose, a microelectrode array was implanted in the spinal dorsal horn of the L6 to the S1 segment. It was demonstrated that the firing rates of the sorted neurons can be used to estimate the pressure^26^ and volume^47^ during the filling of the bladder.

One important issue in decoding continuous action from neural recordings is the decoding model itself. The most common approaches used in the past to monitor the bladder have been linear regression^26,27,36,47^ and Kalman filtering^27^. Despite the existence of efficient linear decoding models, linear regression models only look at the linear relationship between the mean of the dependent variable and the independent variables, while neural signals originate from highly nonlinear and multidimensional systems. Moreover, linear approaches are relatively sensitive to outliers. To improve the performance of the decoding model, some studies used nonlinear regression methods, such as nonlinear autoregressive moving average (NARMA) models^27^ and support vector regression (SVR)^26^. In^27^, it was demonstrated that the NARMA model provided the most accurate bladder pressure estimate (based on the normalized root-mean-squared error) compared to Kalman filtering and linear regression. Moreover, it was shown that the decoding accuracy achieved by SVR was significantly greater than that achieved by the linear filter^26^. However, the nonlinear regression used in^26^ is a static structure in which there is no feedback, and the outputs are calculated directly based on the inputs (i.e., firing rates). The static structure of the decoding method is not an efficient method for representing the nonlinear dynamic relationships between neural signals and bladder states. Moreover, it has difficulty capturing global system behaviors in the identification of nonlinear systems that combine long- and short-term dynamics.

In this paper, we propose a deep recurrent neural network (DRNN) for the estimation of bladder pressure and volume from extracellular neural activity recorded directly from spinal cord gray matter neurons. The model is based on the Long Short-Term Memory (LSTM) architecture, which has emerged as a general and effective model for capturing long-term temporal dependencies^48^. The LSTM is a state-based RNN whose output depends not only on the current information to perform the estimation but also explicitly takes into account the long-term information from the past.

In addition to spiking activity, it has been demonstrated that the local field potential (LFP) activity recorded from the lumbosacral dorsal horn also carries information about intravesical pressure^26^. Neural information processing at each level of observation (spiking, LFP, electroencephalogram) entails the interaction of both evoked (input-driven or stimulus-related) and induced (background processes or intrinsic dynamics) factors. In this paper, we combine modeling of spiking activity and LFP activity into a unified framework to estimate the pressure and volume of the bladder.

Afferents innervating the bladder project to the lumbosacral (L6–S1) segments of the rat spinal cord. The question arises as to which segment provides more information about the bladder. To answer this question, we investigated single-electrode recordings from the L6 and S1 segments using mutual information and decoding performance.

Another important issue in the closed-loop control of the bladder and in developing an efficient therapeutic approach for individuals with spinal cord injury or neurological disorders is the simultaneous measurement of both the pressure and the volume of the bladder. To date, researchers have focused on estimating either the intravesical pressure^26,27^ or volume^36,47^ from neural signals. In this paper, we also investigate the simultaneous estimation of both the pressure and volume of the bladder from single-electrode lumbosacral spinal recording using the proposed decoding model.

## Materials and Methods

### Animal preparation and surgery

**Figure 1:**
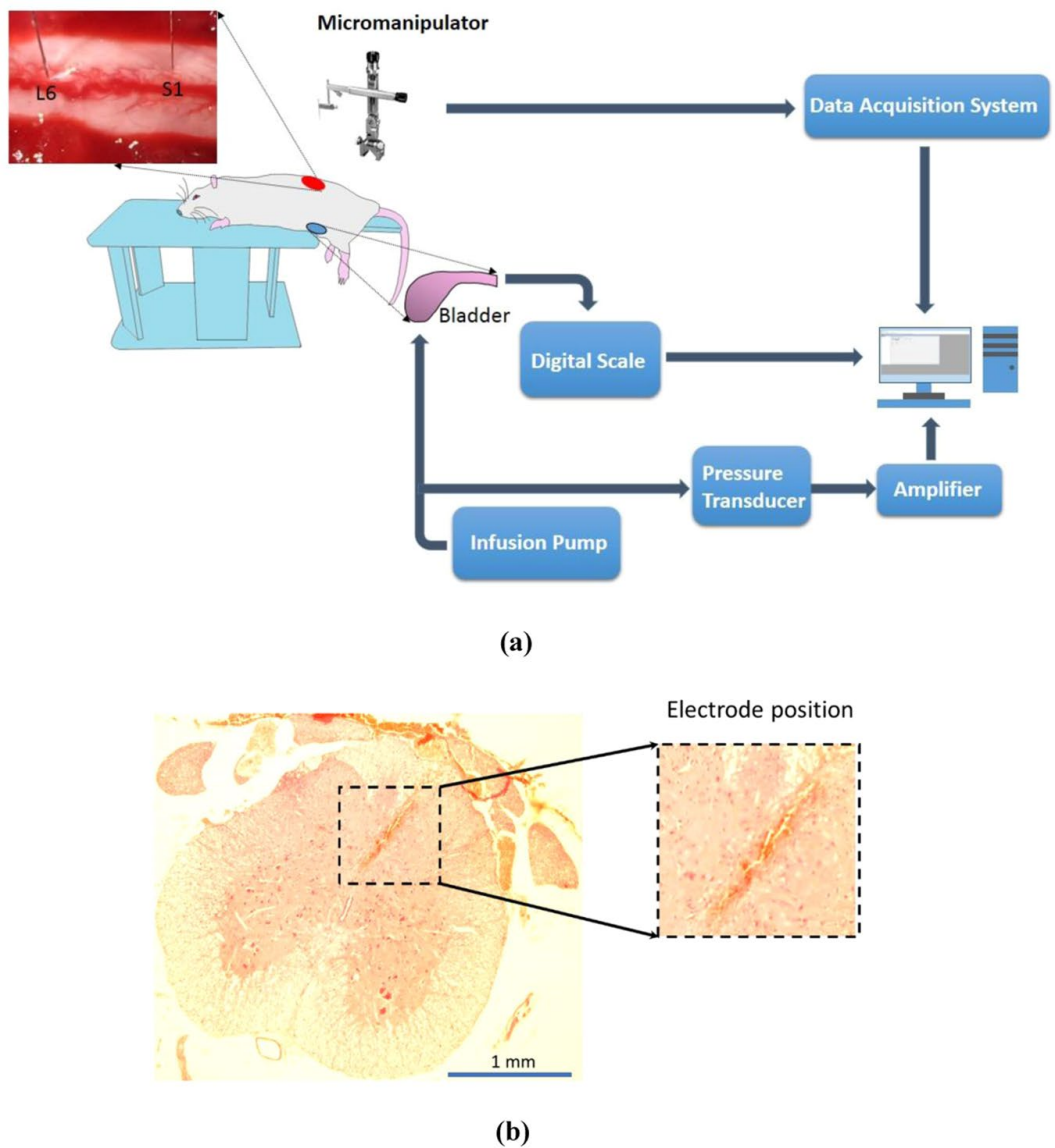
Experimental setup and recording sites. (**a**) A catheter was inserted into the bladder wall thorough the dome and secured with a purse-string suture. One head of the catheter was connected to the pressure transducer and another head to the infusion pump. A digital scale was positioned under the rat to measure the voided volume. One single-needle electrode was inserted into L6, and another one was inserted into the S1 spinal cord segment for neural activity recording. Bladder filling was performed with 0.9% saline at room temperature using an infusion pump at different rates (6, 8, and 10 ml/h). (**b**) A typical histological image from the S1 spinal cord segment of rat 8. Staining was performed with hematoxylin and eosin (H & E).

The experiments were conducted on fifteen intact adult male Wistar rats (180–400 gr). All surgical procedures and experimental protocols involving animal models described in this paper were approved by the Institutional Animal Care and Ethics Committee of Iran Neural Technology Research Center, Iran University of Science and Technology. The whole protocols and methods were performed in accordance with the recommendations and relevant guidelines for the care and use of laboratory animals. The animals were anesthetized with urethane (1.5 g/kg, intraperitoneally) and remained sedated with ketamine (3 mg/kg). The fur around the T13 to the L4 vertebrae was removed. Then, the skin was incised along the vertebrae, and the muscle tissue was removed until the vertebrae were visible. A laminectomy was performed on the lumbar vertebrae (L1 to L2) to expose the L6 to S1 spinal cord segments. To expose the bladder, a ventral midline incision was made. A sterile polyethylene (PE) 50 tube (0.5 mm ID and 0.9 mm OD) (AD Instruments Ltd, Australia) was inserted into the bladder wall through the dome and secured with a purse-string suture. The PE tube was attached to a stopcock connected to a pressure transducer (NovaTrans transducer system, MX860, Smiths Medical ASD, Inc.) and a syringe pump (SN-50C6, Sino Medical-Device Technology Co., Ltd., China) for recording the intravesical bladder pressure and infusing saline into the bladder, respectively. The pressure signals were amplified (900×) and sampled at 50 Hz (maximum sampling rate of pressure sensor is 1 kHz). A digital scale (GF-300, A&D Instruments Ltd., UK) was positioned under the rat to measure the voided volume. The volume signals were sampled at 5 Hz. The experimental setup is illustrated in Fig. 1. The bladder was filled with three different rates of saline solution (i.e., 6, 8, and 10 ml/h), and the neural signals and the pressure and volume of the bladder were recorded simultaneously during filling. Each trial started with the saline infusion and continued until several voiding contractions and bladder leakages occurred. The duration of each trial was between 450 and 850 seconds. At the end of each trial, the bladder was emptied with a syringe, and the rat was relaxed for 20 minutes. During single-electrode recordings, each session of the experiment consisted of four trials per rat. During two-electrode recordings, 2–3 trials of the experiment were conducted per rat. In total, 53 trials were conducted on 15 rats.

### Electrode implantation and neural signal acquisition

Recording electrodes were made from epoxylite-insulated tungsten with shank diameter 75 *μ*m, 5°–10° tapered tip of 120 *μ*m exposed length, and 300–500 kΩ resistance (FHC Inc., Bowdin, ME USA). The microelectrodes were mounted on a micromanipulator (SM-15, Narishige Group Product, Japan) that could control the three-dimensional positioning of the electrode with a minimum graduation of 10 μm. The electrodes were positioned at locations within the L6 and S1 dorsal horn approximately 0–1 mm lateral from the midline between 100 and 200 *μ*m in depth (Fig. 1(b)). To determine the best electrode position within the dorsal horn, the electrode was vertically advanced through the spinal cord dorsoventrally. Then, the electrode was withdrawn and moved 100 *µ*m mediolaterally and/or rostrocaudally to an adjacent location, while the correlation of neural activity with the bladder pressure was visually inspected on the monitor of the recording system. The positions that produced the highest correlations were selected. The recording process was performed within a custom-made Faraday cage to increase the signal-to-noise ratio. Neural signals were amplified (programmable gain, 1000×) and recorded at a 20 kHz sampling rate using a data acquisition system (USB-ME64 system, Multichannel Systems Reutlingen, Germany).

### Preprocessing and feature extraction

The recorded neural signals were bandpass filtered between 300 and 3000 Hz with the low-pass and high-pass elliptic filters of order four. Spikes were detected using the Wave_Clus program, which is publicly available online (http://www2.le.ac.uk/centres/csn/research-2/spike-sorting). The threshold for spike detection was set to four times the standard deviation of the noise estimated from the filtered signal, and spike events were identified as each instance the signal exceeded this threshold. The feature set was formed from the continuous firing rate (FR) and the LFP. All data analyses and decoding models were performed with customized algorithms written in MATLAB.

#### Continuous FR

Continuous FR was computed by taking a Gaussian window of duration 50 ms and counting the number of spikes within the window at each time. Then, the FR was smoothed using a causal moving average filter with a span of 20 samples.

#### Local field potential

The power of the LFP subband component constituted the second feature set. The LFP signals were down sampled to 2000 Hz. Short-time Fourier transform (STFT) was used to generate power spectra over time with a 500 ms Hanning window with 80% overlap. The average of the power spectrum in five frequency bands (1–2.9 Hz, 3–8.8 Hz, 9.1–26.7 Hz, 27.7–81 Hz, 83.9–256 Hz) was used as the neural signal feature for decoding the pressure and the volume of the bladder. The average of the power spectrum was smoothed using a moving average filter with a span of 10 samples. The frequency bands of the LFP signals were selected according to^26^.

### Decoding model

**Figure 2:**
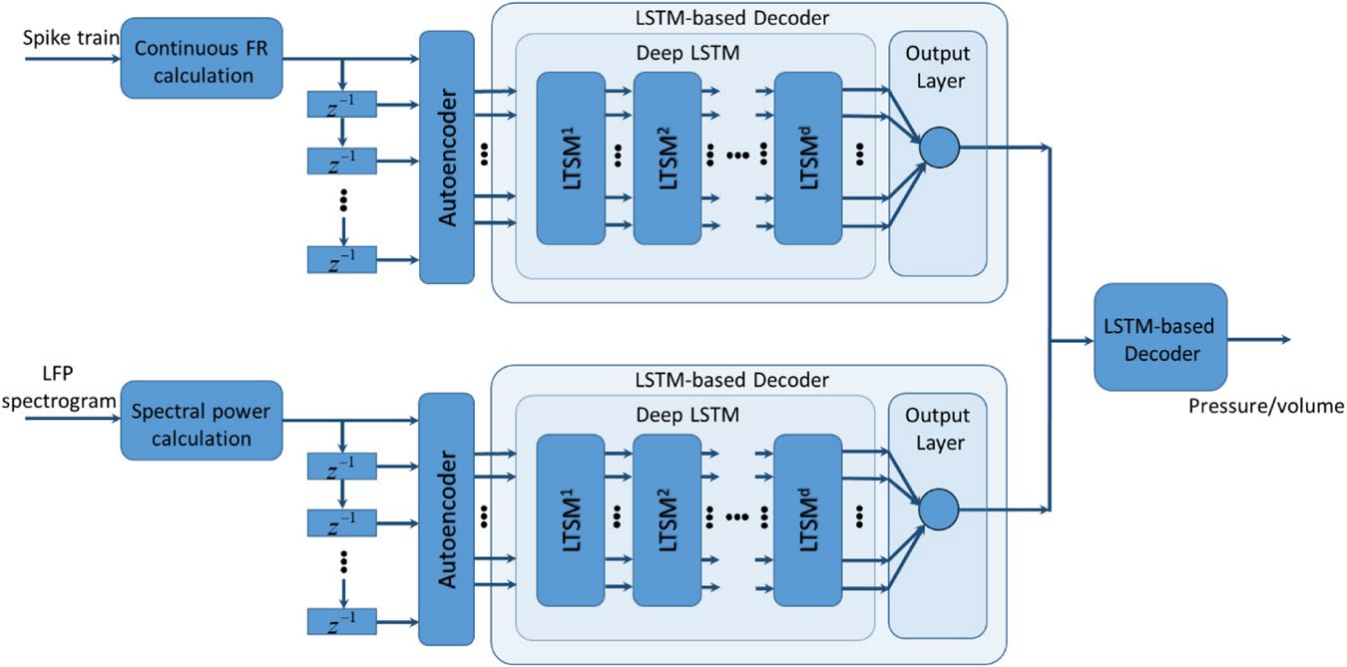
The architecture of the proposed decoding model. The model is based on an autoencoder (AE) and a recurrent neural network with long Short-Term Memory (LSTM).

The architecture of the proposed decoding model is shown in Fig. 2. The model is based on an autoencoder (AE)^49^ and a recurrent neural network with LSTM^50,51^. Autoencoder is one of the deep architecture-based models used to learn low-dimensional features from a high-dimensional input vector^49^. LSTM is a type of RNN that allows learning of long-term temporal dependencies. In principle, the RNN involves dynamic elements in the form of a feedback loop, which feedback the lagged outputs of the neurons to the inputs of neurons^52^. The feedback loops enable the network to perform dynamic mapping and learn tasks that extend over time. However, conventional RNN encounters challenges in modeling long-term dependencies, such as vanishing and exploding gradient problems^48,53^. Recurrent neural networks with LSTM have been shown to be an efficient method to overcome these problems.

By taking advantage of an AE to extract the effective features and an RNN with LSTM to take into account the long-term information in the past, a deep learning structure is proposed for estimating the pressure and volume of the bladder.

### Autoencoder

The basic architecture of an AE network consists of three layers, one input layer, one hidden layer with dimensions less than the dimensions of the input layer, and an output layer with dimensions equal to the dimensions of the input layer^49^. The AE network transforms the high-dimensional input data into a feature space with lower dimensions, while the decoder network can reconstruct the input data from the feature space. The AE attempts to make the network output value close to the input value by minimizing the reconstruction error. After training the AE, the output layer together with its connections from the hidden layer are removed, and the hidden layer is considered the extracted feature. The number of neurons in the input layer and hidden layer are 41 and 30, respectively.

### Deep LSTM

**Figure 3:**
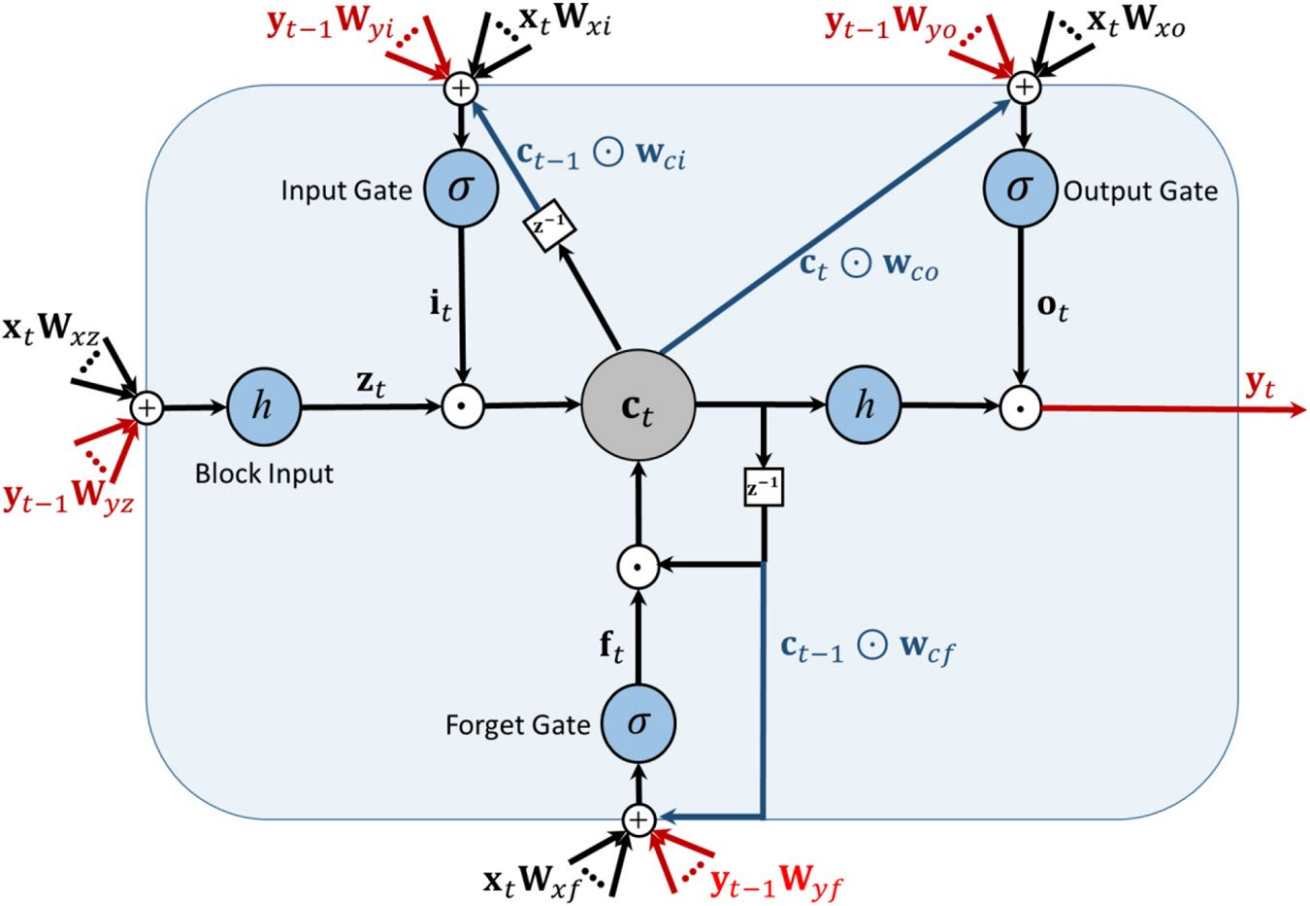
Detailed schematic of a Long Short-Term Memory block (LSTM).

A schematic diagram of the RNN with LSTM is shown in Fig. 3. Each LSTM layer consists of three gates (input, output, forget), block input, block output, memory cell, and peephole connections. The output of the LSTM layer is recurrently connected back to the input layer and to all of the gates of the LSTM layer. The input gate determines the amount of new information entered into the block, the forget gate determines when to forget content regarding the internal state, and the output gate controls the amount of information going to the output. The input, forget, and output gates have a sigmoid activation function, *σ*, and a hyperbolic tangent, *h*, is usually selected as the activation function of the block input and output. We may then describe the dynamic behavior of the network by the following equations:

1 $${{\bf{i}}}_{t}=\sigma ({{\bf{x}}}_{t}{{\bf{W}}}_{xi}+{{\bf{y}}}_{t-1}{{\bf{W}}}_{yi}+{{\bf{w}}}_{ci}\odot {{\bf{c}}}_{t-1}+{{\bf{b}}}_{i})$$

2 $${{\bf{f}}}_{t}=\sigma ({{\bf{x}}}_{t}{{\bf{W}}}_{xf}+{{\bf{y}}}_{t-1}{{\bf{W}}}_{yf}+{{\bf{w}}}_{cf}\odot {{\bf{c}}}_{t-1}+{{\bf{b}}}_{f})$$

3 $${{\bf{z}}}_{t}=h({{\bf{x}}}_{t}{{\bf{W}}}_{xz}+{{\bf{y}}}_{t-1}{{\bf{W}}}_{yz}+{{\bf{b}}}_{z})$$

4 $${{\bf{c}}}_{t}={{\bf{f}}}_{t}\odot {{\bf{c}}}_{t-1}+{{\bf{i}}}_{t}\odot {{\bf{z}}}_{t}$$

5 $${{\bf{o}}}_{t}=\sigma ({{\bf{x}}}_{t}{{\bf{W}}}_{xo}+{{\bf{y}}}_{t-1}{{\bf{W}}}_{yo}+{{\bf{w}}}_{co}\odot {{\bf{c}}}_{t}+{{\bf{b}}}_{o})$$

6 $${{\bf{y}}}_{t}={{\bf{o}}}_{t}\odot h({{\bf{c}}}_{t})$$

where **x** is the input vector to the LSTM, **W**_*xi*_, **W**_*xz*_, **W**_*xf*_, and **W**_*xo*_ are the connection weights from the input to the input gate, block input, forget gate, and output gate, respectively, **W**_*yi*_, **W**_*yz*_, **W**_*yf*_, and **W**_*yo*_ are the recurrent connection weights from the output to the input gate, block input, forget gate, and output gate, respectively, and **w**_*ci*_, **w**_*cf*_, and **w**_*co*_ are the peephole connection weights from the cell gate to the input gate, forget gate, and output gate, respectively. Vectors **b**_*i*_, **b**_*f*_, **b**_*z*_, and **b**_*o*_ are the bias weights determined during training. Symbol $\odot$ represents pointwise multiplication. Three LSTM layers are considered and each LSTM layer contains 10 units. The number of units and the number of LSTM layers are selected heuristically to achieve the best performance.

The AE and LSTM-based decoder was trained using the Levenberg–Marquardt and stochastic gradient descent method, respectively. The Levenberg–Marquardt method is a compromise between the Gradient descent, which has a guaranteed convergence upon a proper choice of the step-size, and Newton’s method, which converges speedily near a local or global minimum.

### Data analysis methods

To assess the performance of the proposed method in estimating bladder pressure and volume, the normalized root-mean-square (NRMS) of the estimation error and the correlation coefficient (CC), were used. The NRMS and CC were defined as

7 $${\rm{NRMS}}( \% )=\frac{1}{{\rm{\max }}({y}_{d}(t))-\,{\rm{\min }}({y}_{d}(t))}\times \sqrt{\frac{1}{T}\mathop{\sum }\limits_{t=1}^{T}{({y}_{d}(t)-y(t))}^{2}}\times 100$$

8 $${\rm{CC}}( \% )=(\frac{{\sum }_{t=1}^{T}(y(t)-\bar{y})({y}_{d}(t)-{\bar{y}}_{d})}{{\sum }_{t=1}^{T}{(y(t)-\bar{y})}^{2}\,{\sum }_{t=1}^{T}{({y}_{d}(t)-{\bar{y}}_{d})}^{2}})\times 100$$

where *y*_*d*_(*t*) is the desired pressure or volume and *y*(t) is the estimated value of desired pressure or volume. Moreover, to evaluate the information content of the neural signals recorded from different vertebral segments, the mutual information (MI) was calculated between the neural signal feature of interest (firing rate or LFP signal band power spectrum) of each vertebral segment and the bladder parameter of interest (pressure or volume). Mutual information was calculated based on an adaptive partitioning of the observation space^54^. Two-way analysis of variance (ANOVA) was used to assess the statistical significance of the results and differences, and a confidence level of 95% (*p* < 0.05) was chosen to indicate a significant difference.

Three methods were used for evaluating the proposed method: inside-trial, trial-by-trial, and rat-by-rat. For the inside-trial evaluation, the deep LSTM was trained and tested with the data obtained during each trial of experiment. In this case, 70% of the data were used for training, and the remaining 30% of the data were used for testing. For the trial-by-trial evaluation, training was performed on the data obtained during one trial of the experiment and tested with the remaining trials. For the rat-by-rat evaluation method, the deep LSTM was trained with one trial of the experiments on one rat and tested with the data obtained from each rat. In total, 53 trials were conducted on 15 rats. The duration of each trial was between 450 and 850 seconds.

## Results

**Figure 4:**
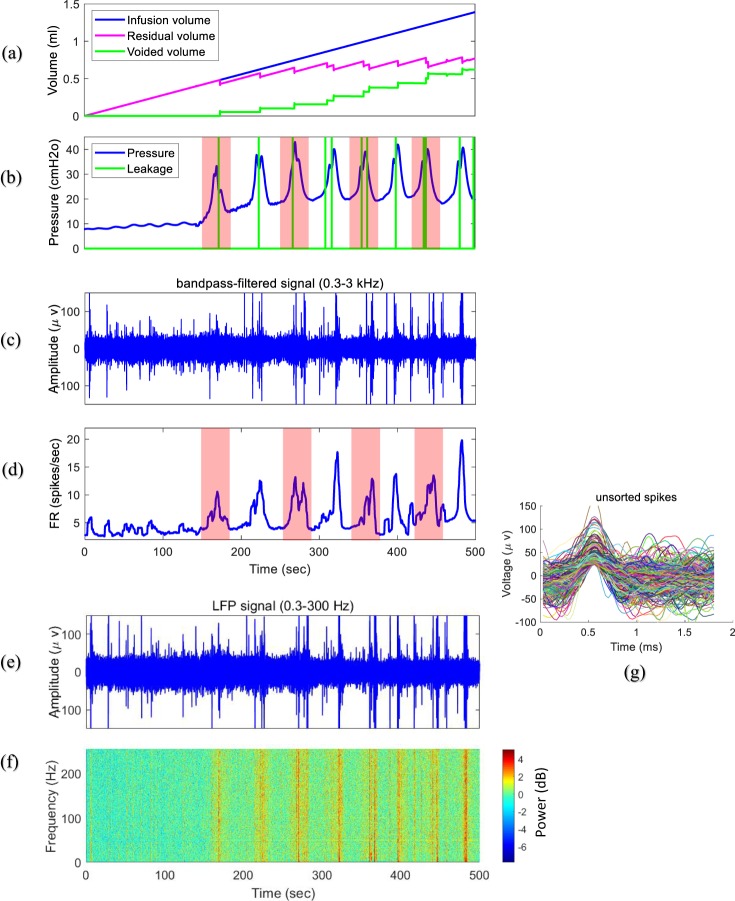
Typical signals recorded from the bladder (rat 5, trial 3, infusion rate = 10 ml/h). (**a**) Infusion volume, residual volume, and voided volume. (**b**) Bladder pressure and leakage events. (**c**) Bandpass filtered (0.3–3 kHz) neural signal from S1. (**d**) Continuous firing rate of the detected spikes. (**e**) Recorded local field potential (*LFP*) signal. (**f**) Time-frequency analysis of the LFP signal. (**g**) Unsorted detected spike waveforms.

Figure 4 shows the recorded infused volume, residual volume, voided volume, bladder pressure, bandpass filtered neural signal (0.3–3 kHz), continuous firing rate, intraspinal LFP activity and corresponding time-frequency analysis during a typical trial from the experiment (rat 5, trial 3, infusion rate = 10 ml/h). It is observed that both the bladder pressure and the firing rate monotonically increase with increasing bladder volume. When the bladder pressure suddenly increases, bladder leakage occurs.

**Figure 5:**
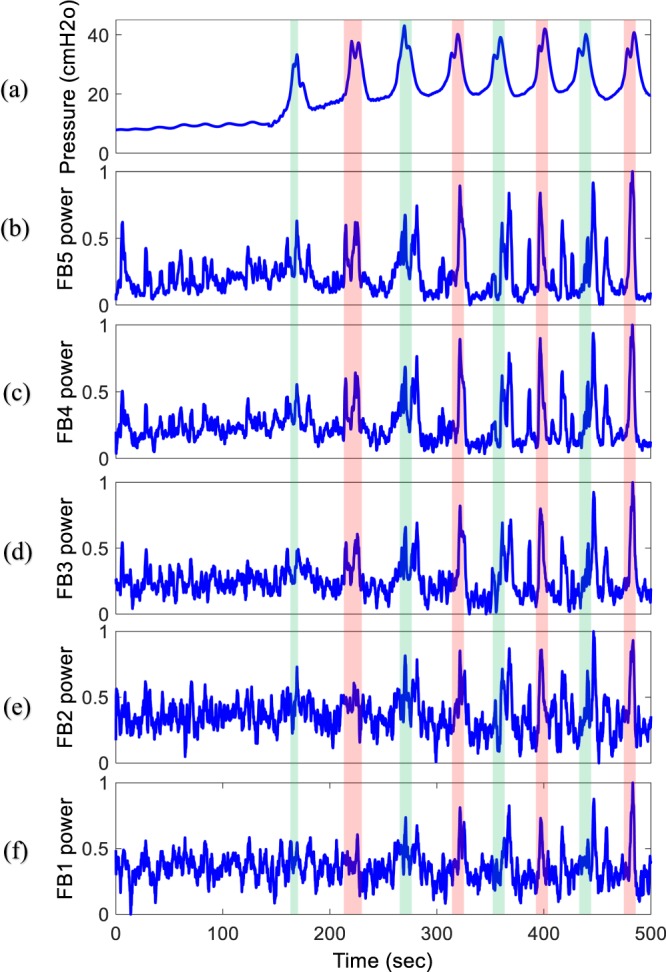
An example of the bladder pressure and power spectrum of the recorded LFP signal recorded from S1 (trial 3, rat 5, infusion rate = 10 ml/h) in different frequency bands: FB5: 83.9–256 Hz, FB4: 27.7–81 Hz, FB3: 9.1–26.7 Hz, FB2: 3–8.8 Hz, FB1: 1–2.9 Hz. Colored vertical bars are provided to help visualize changes in frequency bands across the subplots as the bladder pressure changes.

The time-frequency analysis of the intraspinal LFP shows that spectral patterns could clearly reflect the status of the bladder. Figure 5 shows the typical frequency bands of the intraspinal LFP signal during bladder filling. The power of the defined frequency bands suddenly increases as the bladder pressure increases, and the local maxima of the LFP frequency bands show the instant when bladder leakage occurs.

**Figure 6:**
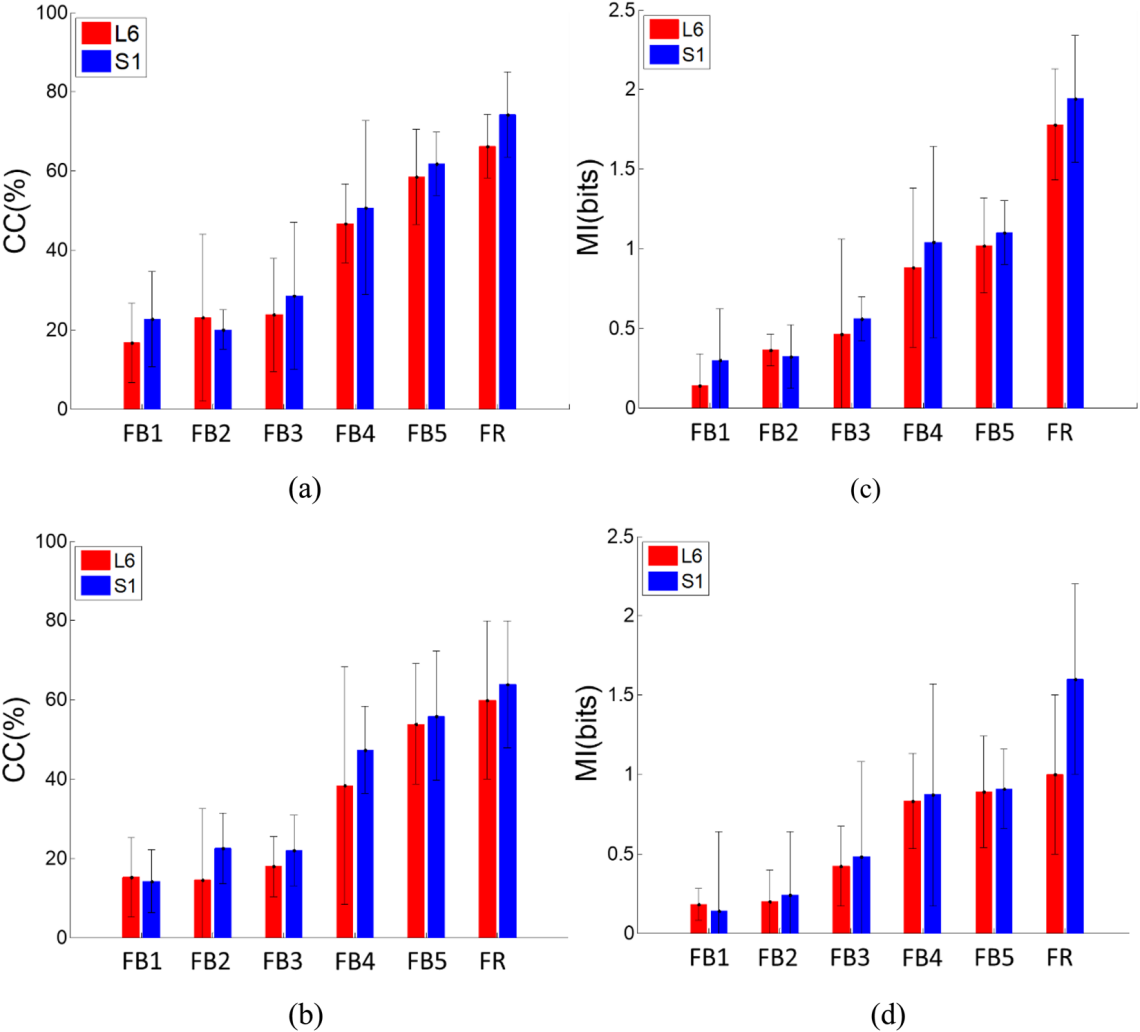
Correlation coefficient and mutual information between bladder statuses with extracted features. (**a**) Correlation coefficient between bladder pressure and extracted features. (**b**) Correlation coefficient between volume and extracted features. (**c**) Mutual information between pressure and extracted features. (**d**) Mutual information between volume and extracted features. Error bars indicate standard deviations.

To assess the information content in the frequency bands of intraspinal LFP signals and in the continuous firing rate with respect to bladder pressure and volume, the averages of the CC and mutual information for 40 trials of experiment on 10 rats were computed (Fig. 6).

The results show that the firing rate provides more information about bladder pressure and volume (*p* < 0.05 based on both CC and MI. The comparison was performed between the mutual information and CC obtained from both the frequency bands and the FR. It can be seen that the neural signals recorded from S1 contain significantly more information about bladder pressure than those recorded from L6 (*p* = 0.013 for CC and *p* = 0.01 for MI) but that there was no significant difference in the information between S1 and L6 with respect to volume (*p* = 0.0836 for MI and *p* = 0.429 for CC). Moreover, the results of the statistical test show that the high frequency components of the LFP signals provide a higher correlation and greater information than the low frequency components (*p* < 0.05) with respect to both bladder pressure and volume. Comparisons were performed between low frequency bands (FB1, FB2, FB3, and FB4) and high frequency bands (FB5) using CC as well as MI.

### Single electrode decoding

**Figure 7:**
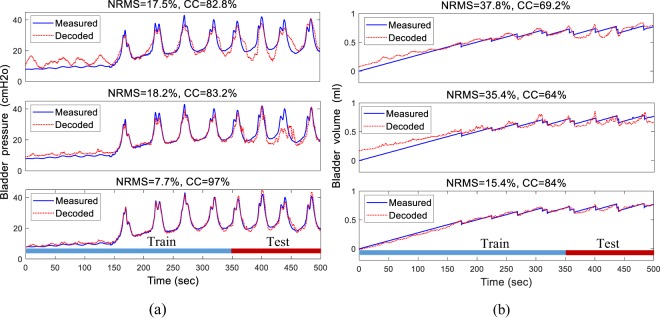
An example of decoding the bladder pressure (**a**) and volume (**b**) using an inside-trial validation approach (training with 70% of trial 3 and testing with the remaining 30% of the same trial) on rat 5 (infusion rate = 10 ml/h). Decoding was performed using the signal recorded from S1 for different extracted feature sets: firing rate (upper trace), FB5 subband power spectrum of the LFP (middle trace), and the combined FR and FB5 subband power spectrum (lower trace). The NRMS and CC values given refer to the test period.

In this section, the performance in decoding bladder pressure/volume using signals recorded from the L6 segment and the S1 segment are presented. Figure 7 shows a typical decoding of the pressure/volume during one trial of the experiment (rat 5, trial 3, infusion rate = 10 ml/h) using FR, LFP band power spectra, and a combination of FR and LFP band power spectra. It is observed that excellent decoding performance is obtained using the combination of FR and LFP band power spectra. The decoding errors are 7.7% and 15.4% for pressure and volume, respectively.

Table 1 summarizes the average inside-trial decoding performance obtained using the neural signals recorded from the L6 segment and the S1 segment for 40 trials of experiments on 10 rats. For both pressure and volume estimation, the decoding performance obtained using the combined FR and LFP data was significantly better than that obtained using FR or LFP alone (*p* < 0.001 for both NRMS and CC). Moreover, the CC shows that the S1 signals provided significantly better decoding performance than those of the L6 (*p* < 0.0013).

**Table 1:** Average and standard deviation of decoding performance across 10 rats using the inside-trial validation approach using single-electrode recording from S1 and L6 segments.

		S1 segment		L6 segment	
		NRMS%	CC%	NRMS%	CC%
FR	pressure	22.5 ± 4.8	72.5 ± 12.4	23.6 ± 5.1	69.7 ± 8.1
	volume	28.2 ± 6.9	60.8 ± 13.4	30.2 ± 4.7	56.3 ± 10.3
LFP	pressure	24.6 ± 8.9	66.6 ± 14.2	26.8 ± 5.0	64.0 ± 13.3
	volume	32.4 ± 5.1	55.2 ± 12.8	35.6 ± 5.3	48.4 ± 12.0
FR & LFP	pressure	18.3 ± 6.7	78.2 ± 18.2	20.7 ± 9.4	75.0 ± 12.4
	volume	24.2 ± 10.9	68.2 ± 17.0	27.8 ± 5.4	62.2 ± 10.0

**Figure 8:**
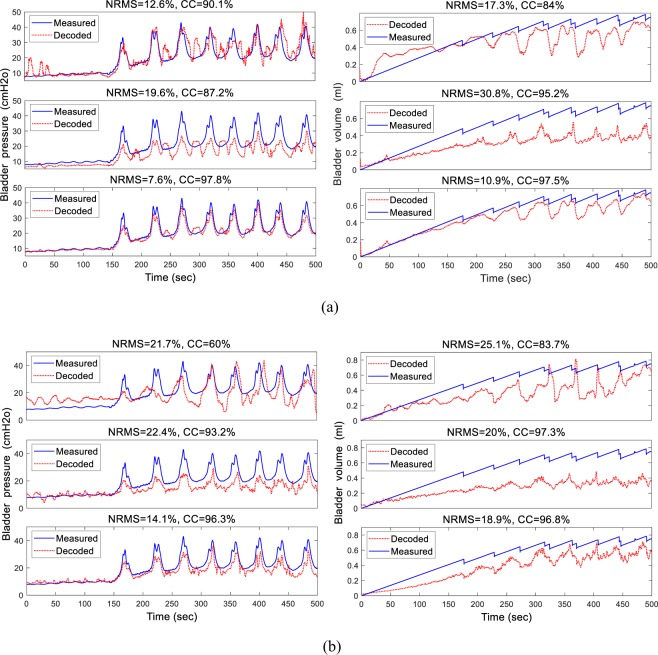
An example of decoding the bladder pressure (left) and volume (right) using trial-by-trial (**a**) and rat-by-rat (**b**) validation approaches. Decoding was performed using different extracted feature sets: firing rate (upper trace), FB5 subband power spectrum of the LFP (middle trace), and the combination of FR and FB5 subband power spectrum (lower trace). In this example, during the trial-by-trial validation approach, the network was trained with trial 1 from rat 5 (infusion rate = 8 ml/h) and tested with trial 3 from the same rat (infusion rate = 10 ml/h). During the rat-by-rat validation approach, the network was trained with rat 7 (trial 1, infusion rate = 8 ml/h) and tested with rat 5 (trial 3, infusion rate = 10 ml/h).

Figure 8(a) shows the result of a typical decoding of pressure and volume using the trial-by-trial validation approach. Training was performed with rat 5, trial 1 with an infusion rate of 8 ml/h, and testing was performed on the same rat, trial 3 with an infusion rate of 10 ml/h. The NRMS decoding errors for pressure decoding were 19.6%, 12.6%, and 7.6% using LFP, FR, and both LFP and FR, respectively, and 30.8%, 17.3%, and 10.9%, respectively, for volume decoding. The CC values for pressure decoding were 87.2%, 90.1%, and 97.8% using LFP, FR, and combined LFP and FR, respectively, and 95.2%, 84.0%, and 97.5%, respectively, for volume decoding. The results indicate that the combination of LFP and FR provided better performance than using only LFP or FR in terms of both NRMS and CC indices. The decoding performance from single-electrode recording from the S1 segments of 10 rats using trial-by-trial validation are summarized in Table 2. The average pressure decoding errors were 26.5 ± 5.0%, 22.3 ± 5.2%, and 17.8 ± 4.8% using LFP, FR, and the combination of LFP and FR, respectively, and 30.0 ± 6.3%, 25.4 ± 3.5%, and 21.3 ± 4.5%, respectively, for volume decoding. Additionally, the average CCs for pressure decoding were 66.0 ± 8.0%, 74.2 ± 6.5%, 80.3 ± 7.3% using LFP, FR, and the combination of LFP and FR, respectively, and 60.9 ± 13.1%, 65.3 ± 7.8%, and 70.5 ± 9.9%, respectively, for volume decoding. The results for pressure decoding show that the combined LFP and FR provided significantly better performance than LFP or FR alone (*p* < 0.001 for NRMS and CC). The results show that the decoding error obtained for volume using the combined LFP and FR was significantly less than that obtained using only LFP or FR (*p* = 0.0011 for NRMS). Moreover, the CC shows that the decoding performance obtained using the combined LFP and FR was better than that obtained using only LFP or FR, but the difference was not statistically significant (*p* = 0.1169).

**Table 2:** Average (mean ± standard deviation) of the decoding performance across 10 rats with single-electrode recording from S1 using the trial-by-trial validation approach.

	LFP		FR		LFP & FR	
	pressure	volume	pressure	volume	pressure	volume
NRMS%	26.5 ± 5.0	30 ± 6.3	22.3 ± 5.2	25.4 ± 3.5	17.8 ± 4.8	21.3 ± 4.5
CC%	66.0 ± 8.0	60.9 ± 13.1	74.2 ± 6.5	65.3 ± 7.8	80.3 ± 7.3	70.5 ± 9.9

Figure 8(b) shows an example of decoding using rat-by-rat validation. Training was performed on rat 7, trial 1, with an infusion rate of 8 ml/h, and testing was performed on rat 5, trial 3 with an infusion rate of 10 ml/h. The NRMS decoding errors obtained were 22.4%, 21.7%, and 14.1% using LFP, FR, and both LFP and FR, respectively, and 20.0%, 25.1%, and 18.9%, respectively, for volume decoding. The CC values for pressure decoding were 93.2%, 60.0%, and 96.3% using LFP, FR, and combined LFP and FR, respectively, and 97.3%, 83.7%, and 96.8%, respectively, for volume decoding. The results show that the decoding model is robust with respect to the training rat. The results of the decoding performance using the rat-by-rat validation approach are summarized in Table 3. In this approach, the data obtained during one trial from one rat were used to train the model, and the data obtained during all trials from all rats were used to test the model. The results indicate that using the combined FR and LFP provides significantly better performance than using only FR or LFP for both pressure and volume (*p* = 0.0033 for pressure and *p* = 0.0015 for volume using NRMS). The results show that the training data obtained from different rats have no significant effect on the decoding performance (*p* = 0.3140 for pressure and *p* = 0.2596 for volume using NRMS).

**Table 3:** Average (mean ± standard deviation) of the decoding performance across 10 rats with single-electrode recording from S1 using the rat-by-rat validation approach.

		Train Rat 1		Train Rat 3		Train Rat 5		Train Rat 7	
		NRMS (%)	CC (%)	NRMS (%)	CC (%)	NRMS (%)	CC (%)	NRMS (%)	CC (%)
Pressure	LFP	26.2 ± 9.2	62.4 ± 10.5	28.9 ± 6.9	58.9 ± 5.4	26.3 ± 8.4	63.4 ± 6.5	27.6 ± 9.1	61.0 ± 13.9
	FR	23.5 ± 8.1	66.3 ± 9.6	26.7 ± 7.2	62.5 ± 9.6	22.9 ± 9.0	69.2 ± 9.9	25.9 ± 7.3	65.3 ± 12.5
	FR&LFP	21.0 ± 4.7	71.7 ± 8.3	23.0 ± 9.4	68.8 ± 7.4	20.2 ± 5.1	73.0 ± 7.9	22.4 ± 6.9	70.0 ± 10.3
Volume	LFP	29.5 ± 6.4	56.7 ± 13.4	30.2 ± 9.8	54.5 ± 9.2	28.2 ± 8.8	57.3 ± 6.7	31.0 ± 5.7	54.5 ± 15.5
	FR	26.9 ± 6.7	60.3 ± 10.8	28.2 ± 8.2	57.8 ± 8.3	24.8 ± 9.7	62.2 ± 8.1	28.1 ± 9.9	57.8 ± 11.3
	FR&LFP	23.0 ± 3.5	65.7 ± 5.2	25.0 ± 4.1	60.5 ± 6.1	22.9 ± 4.6	67.3 ± 8.7	25.0 ± 4.8	62.7 ± 10.1

### Two-electrode decoding

**Figure 9:**
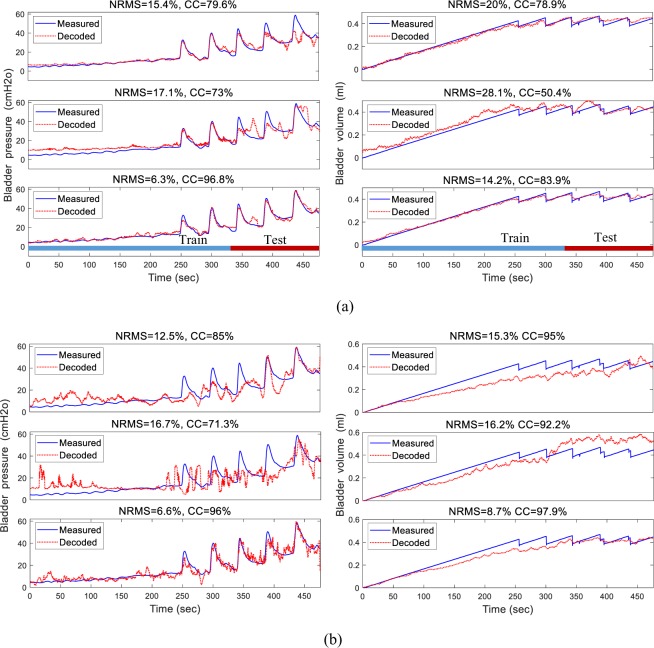
An example of decoding the bladder pressure (left) and volume (right) during two-electrode recording using inside-trial (**a**) and trial-by-trial (**b**) validation approaches (rat 13, trial 2, infusion rate = 6 ml/h). Decoding was performed using both FR and the FB5 subband power spectrum extracted from neural signals recorded from S1 (upper trace), L6 (middle trace), and both S1 and L6 (lower trace). During the trial-by-trial validation approach, the network was trained with trial 1 (rat 13, infusion rate = 6 ml/h) and tested with trial 2 on the same rat (infusion rate = 6 ml/h).

In two-electrode decoding, two electrodes were implanted into the S1 and L6 spinal cord segments, and the compound signals recorded from both segments were used for decoding. Figure 9 shows a typical pressure and volume decoding using the signal recorded from S1, L6, or the combined signal from S1 and L6 during two-electrode recording. Figure 9(a) illustrates the inside-trial decoding results. The NRMS values obtained for pressure decoding were 15.4%, 17.1%, and 6.3% using S1, L6, and the combined signals from S1 and L6, respectively, and 20.0%, 28.1%, and 14.2%, respectively, for volume decoding. The CC values for pressure decoding were 79.6%, 73.0%, and 96.8% using S1, L6, and the combined signals from L6 and S1, respectively, and 78.9%, 50.4%, and 83.9%, respectively, for volume decoding. Figure 9(b) shows the trial-by-trial decoding results. The NRMS pressure decoding errors obtained were 12.5%, 16.7% and 6.6% using S1, L6, and the combined signals recorded from S1 and L6, respectively. Compared to the single-electrode decoding, the two-electrode decoding yielded 47.2% and 60.5% improvement using S1 and L6, respectively. The NRMS volume decoding errors obtained were 15.3%, 16.2% and 8.7% using the signals recorded from S1, L6, and the combined signal from S1 and L6, respectively. Using two-electrode decoding, an improvement of 43.1% and 46.3% was achieved when compared with single-electrode decoding using the signals from S1 and L6, respectively.

Table 4 summarizes the results of decoding performance for five rats during single-electrode and two-electrode decoding using the combined LFP and FR signals. In this analysis, the trial-by-trial validation approach was used. The results show that the average NRMS decoding errors for pressure and volume decoding were 17.6 ± 2.7% and 22.7 ± 3.4%, respectively, when using the S1 signal, 19.8 ± 2.4% and 27.2 ± 4.6%, respectively, when using the L6 signal, and 14.9 ± 4.5% and 19.7 ± 4.7%, respectively, when the combined signal recorded from S1 and L6 was used for decoding. On average, using two-electrode decoding, an improvement of 21.5% and 20.4% was achieved for pressure and volume estimation, respectively, over single-electrode decoding. Almost the same results were observed for the CC. The results of the statistical test show that the two-electrode decoding achieved a lower NRMS error than single-electrode decoding (*p* = 0.0318 for pressure and *p* = 0.0082 for volume).

**Table 4:** Average (mean ± standard deviation) of NRMS and CC from two-electrode recoding using the trial-by-trial validation approach.

Animal	Pressure/Volume	L6		S1		L6 & S1	
		NRMS (%)	CC (%)	NRMS (%)	CC (%)	NRMS (%)	CC (%)
Rat 11	pressure	18.7 ± 0.3	75.5 ± 2.1	18.9 ± 4.3	78.0 ± 1.4	12.8 ± 0.7	80.7 ± 2.4
	volume	30.1 ± 2.6	61.8 ± 0.07	24.0 ± 3.5	69.2 ± 1.7	20.0 ± 2.0	70.8 ± 4.7
Rat 12	pressure	22.0 ± 2.8	74.7 ± 1.9	18.0 ± 1.3	79.7 ± 0.7	17.9 ± 0.3	80.1 ± 1.9
	volume	28.0 ± 0.7	62.2 ± 0.8	23.1 ± 0.7	65.5 ± 2.7	20.5 ± 0.2	69.1 ± 1.6
Rat 13	pressure	19.4 ± 3.8	73.1 ± 2.6	15.5 ± 4.2	81.0 ± 5.6	12.3 ± 1.4	88.1 ± 11.1
	volume	22.1 ± 8.4	78.6 ± 19.2	18.7 ± 4.8	80.0 ± 21.2	14.5 ± 8.2	84.6 ± 18.5
Rat 14	pressure	18.8 ± 1.1	69.8 ± 6.1	18.1 ± 0.7	76.6 ± 1.9	15.8 ± 1.8	82.9 ± 0.4
	volume	27.5 ± 0.8	58.8 ± 4.7	24.4 ± 1.6	68.7 ± 3.5	21.3 ± 3.0	75.6 ± 3.3
Rat 15	pressure	20.1 ± 1.6	75.6 ± 0.6	17.7 ± 1.3	78.8 ± 0.3	15.7 ± 1.7	84.6 ± 0.9
	volume	28.5 ± 0.4	51.0 ± 0.6	24.0 ± 0.6	67.1 ± 0.5	22.6 ± 0.6	71.0 ± 0.6
Average	pressure	19.8 ± 2.5	73.7 ± 3.8	17.6 ± 2.6	78.8 ± 2.3	14.9 ± 4.8	83.2 ± 3.2
	volume	27.2 ± 4.7	62.4 ± 13.5	22.8 ± 3.5	70.1 ± 9.9	19.7 ± 4.7	74.2 ± 6.2

### Signal stability

**Figure 10:**
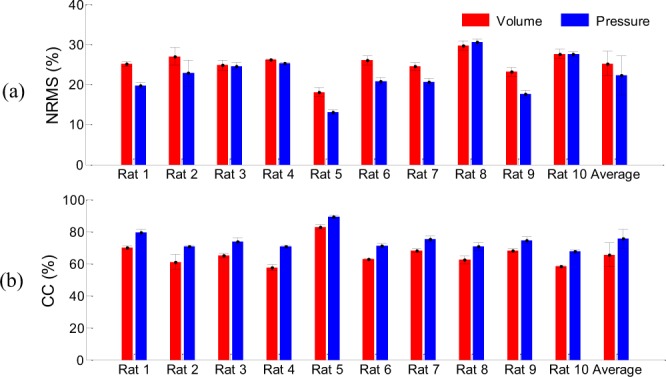
Decoding performance for each rat using the trial-by-trial validation approach. (**a**) NRMS errors for volume (red) and pressure (blue) estimation. (**b**) Correlation coefficients for volume (red) and pressure (blue) estimation.

Figure 10 shows the decoding performance for each rat using a trial-by-trial validation approach for single electrode recording. The standard deviation for each rat is very small, which indicates the stability of the decoding across trials.

## Discussion

In this paper, we propose a DRNN for the estimation of bladder pressure and volume from neural activity recorded directly from spinal cord gray matter neurons. The proposed DRNN consists of an autoencoder and a deep LSTM-based decoder. The autoencoder has a deep structure that extracts deep features from input data in an unsupervised manner^49^. The decoding model is based on the LSTM, which has emerged as a general and effective model for capturing long-term temporal dependencies^48^. The LSTM consists of memory cells whose inputs and outputs are controlled by nonlinear gates. Its output not only depends on the current information to perform the estimation but also explicitly takes into account the long-term information from the past.

Since LFP reflects a spatial averaging of synaptic activity in the vicinity of the electrode and carries information that is distinct from spikes, in this paper, we combined modeling of both spiking activity and LFP activity in a unified framework to estimate the pressure and volume of the bladder. The results show that using a combination of FR and LFP can improve decoding performance with respect to using FR or LFP alone.

An important issue in estimating bladder status using neural signals recorded from the spinal cord is the selection of spinal segment that provides more information about the bladder. Afferents innervating the bladder project to the lumbosacral (L6–S1) segments of the rat spinal cord. To address this issue, single-electrode recordings from the L6 and S1 segments were investigated using mutual information and decoding performance. The results show that the S1 segment provides more information about the bladder status than the L6 segment.

Another issue investigated in this study was the effect of two-electrode recoding on decoding performance. For this purpose, a combined signal recorded simultaneously from S1 and L6 was used for decoding, and the results were compared with single-electrode decoding. The results show that the combined signal from both segments provided significantly better decoding performance than the single-electrode decoding. Using an array of electrodes implanted along the L6-S1 segments may improve the decoding performance further. Our results show that average CCs of 78.8 ± 2.3% and 83.2 ± 3.2% were obtained for pressure estimation using single-electrode and two-electrode decoding, respectively. In contrast, the results of pressure estimation presented in^26^ show that increasing the number of recording channels did not increase the decoding performance. In aforementioned paper, the CC reported for one channel was approximately 75.0 ± 15.3%, while for 8 channels it was 75.5 ± 14.6%, using SVR with 30 filter taps. This lack of enhancement may be related to the nonoptimal design of the filter and improper positioning of the electrode tips. Their results show that a CC of approximately 81.8 ± 10.0% was obtained for bladder pressure estimation using SVR with 4 channels and 100 filter taps. Regardless of decoding performance, to obtain chronic stability from the spinal recoding, it is necessary to use a multi-electrode array rather than a one- or two-electrode recording system.

An important contribution of the current study is the simultaneous estimation of both the pressure and the volume of the bladder. Simultaneously estimating both the pressure and the volume of the bladder is a critical issue in the closed-loop control of the bladder using functional electrical stimulation. The results illustrate that utilizing the proposed deep neural network, the simultaneous estimation of bladder volume and pressure is possible, which had not been investigated in previous studies.

Another issue investigated in this paper is the estimation of pressure/volume for different infusion rates. While the individual is conscious, the bladder filling rate may vary according to different conditions. Different infusion rates lead to different rise times of the bladder pressure (slower or faster). Hence, the decoding model should be generalizable to new conditions. The results of this study show that the proposed deep model is able to estimate the pressure/volume of the bladder for different infusion rates.

The proposed method in this paper has provided very promising results in animal models, but a number of challenges will have to be addressed to establish the clinical use of the method. One of the limitations of the current study is the invasiveness of the measurements, which presents challenges in translating the intraspinal recordings from animal studies to clinical practice. Implanting microelectrodes into the spinal cord can carry the risk of infection and spinal compression. Another challenge facing intraspinal recording is the fabrication and implantation of intraspinal microelectrodes. However, recent progress in microelectrode arrays^55–62^ and knowledge gained from implantable electrodes on animals can be applied to the development of a chronically implanted prosthetic device. Nevertheless, before clinical use of spinal recoding, further tests will be required to assess the long-term mechanical stability of chronically implanted electrodes and the longevity of the chronic recordings. In addition, accurate placement of electrodes in the spinal cord is challenging, even using intraoperative guidance for electrode implantation.

Another critical issue in estimating bladder status using neural signals recorded from the spinal cord is interference from non-bladder signals. It is expected that the decoding model could extract the relevant information about the bladder pressure/volume from neural activity during learning and filter non-bladder signals. However, we did not justify this filtering in this study; this analysis could be considered for a future study. Additionally, further studies should be performed to test the performance of the proposed decoding model in real time for closed-loop control of the bladder.

## Data Availability

All datasets generated during the current study are available from the corresponding author upon request.

## References

[1] Fowler CJ, Griffiths D, Groat WCD (2008). The neural control of micturition. Nat. Rev. Neurosci..

[2] McGee MJ, Amundsen CL, Grill WM (2015). Electrical stimulation for the treatment of lower urinary tract dysfunction after spinal cord injury. J. Spinal Cord Med..

[3] Chen G, Liao L, Dong Q, Ju Y (2012). The inhibitory effects of pudendal nerve stimulation on bladder overactivity in spinal cord injury dogs: is early stimulation necessary?. Neuromodulation: Technology at the Neural Interface..

[4] McGee MJ, Amundsen CL, Grill WM (2016). Temporal pattern of stimulation modulates reflex bladder activation by pudendal nerve stimulation. Neurourol..

[5] Wenzel BJ, Boggs JW, Gustafson KJ, Grill WM (2006). Closed loop electrical control of urinary continence. J. Urol..

[6] Opisso E, Borau A, Rijkhoff NJM (2011). Urethral sphincter EMG-controlled dorsal penile/clitoral nerve stimulation to treat neurogenic detrusor overactivity. J. Neural Eng..

[7] Bhadra N, Bhadra N, Kilgore K, Gustafson KJ (2006). High frequency electrical conduction block of the pudendal nerve. J. Neural Eng..

[8] Tai C, Wang J, Wang X, Roppolo JR, Groat WC (2007). Voiding reflex in chronic spinal cord injured cats induced by stimulating and blocking pudendal nerves. Neurourol. Urodyn: Official Journal of the International Continence Society..

[9] Boger A, Bhadra N, Gustafson KJ (2008). Bladder voiding by combined high frequency electrical pudendal nerve block and sacral root stimulation. Neurourol Urodyn: Official Journal of the International Continence Society..

[10] Boger AS, Bhadra N, Gustafson KJ (2012). High frequency sacral root nerve block allows bladder voiding. Neurourol..

[11] Peh WYX (2018). Novel neurostimulation of autonomic pelvic nerves overcomes bladder-sphincter dyssynergia. Front. Neurosci..

[12] Kirkham APS, Shah NC, Knight SL, Shah PJR, Craggs MD (2001). The acute effects of continuous and conditional neuromodulation on the bladder in spinal cord injury. Spinal cord..

[13] Dalmose AL (2003). Conditional stimulatzion of the dorsal penile/clitoral nerve may increase cystometric capacity in patients with spinal cord injury. Neurourol..

[14] Hansen J (2005). Treatment of neurogenic detrusor overactivity in spinal cord injured patients by conditional electrical stimulation. J. Urol..

[15] Horvath EE, Yoo PB, Amundsen CL, Webster GD, Grill WM (2010). Conditional and continuous electrical stimulation increase cystometric capacity in persons with spinal cord injury. Neurourol Urodyn: Official Journal of the International Continence Society..

[16] Brouillard CB, Crook JJ, Irazoqui PP, Lovick TA (2018). Suppression of urinary voiding by conditional high frequency stimulation of the pelvic nerve in conscious rats. Front. Physiol..

[17] Melgaard J, Rijkhoff NJM (2011). Detecting the onset of urinary bladder contractions using an implantable pressure sensor. IEEE Trans. Neural syst. Rehabil. Eng..

[18] Majerus SJ, Fletter PC, Damaser MS, Garverick SL (2011). Low-power wireless micromanometer system for acute and chronic bladder-pressure monitoring. IEEE Trans. Biomed. Eng..

[19] Lee DS (2011). Real-time bladder volume monitoring by the application of a new implantable bladder volume sensor for a small animal model. Kaohsiung J. Med. Sci..

[20] Lee HY (2016). Sensitivity-enhanced LC pressure sensor for wireless bladder pressure monitoring. IEEE Sens. J..

[21] Majerus SJ (2017). Suburothelial bladder contraction detection with implanted pressure sensor. PloS one..

[22] Weber MJ (2018). A Miniaturized single-transducer implantable pressure sensor with time-multiplexed ultrasonic data and power links. CIRC..

[23] Stauffer F (2018). Soft electronic strain sensor with chipless wireless readout: toward real‐time monitoring of bladder volume. Adv. Mater. Technol..

[24] Abelson B (2019). Ambulatory urodynamic monitoring: state of the art and future directions. Nat. Rev. Urol.

[25] Yu L, Kim B, Meng E (2014). Chronically implanted pressure sensors: challenges and state of the field. Sensors..

[26] Im C (2016). Decoding intravesical pressure from local field potentials in rat lumbosacral spinal cord. J. Neural Eng..

[27] Ross SE, Ouyang Z, Rajagopalan S, Bruns TM (2018). Evaluation of decoding algorithms for estimating bladder pressure from dorsal root ganglia neural recordings. ANN. Biomed. Eng..

[28] Hansen J (2007). Urethral sphincter EMG as event detector for neurogenic detrusor overactivity. IEEE Trans. Biomed. Eng..

[29] Knight SL, Edirisinghe N, Leaker B, Susser J, Craggs MD (2018). Conditional neuromodulation of neurogenic detrusor overactivity using transrectal stimulation in patients with spinal cord injury: A proof of principle study. Neurourol..

[30] Rutter EM (2018). Detection of bladder contractions from the activity of the external urethral sphincter in rats using sparse regression. IEEE Trans. Neural Syst. Rehabil. Eng..

[31] Wenzel BJ, Boggs JW, Gustafson KJ, Grill WM (2005). Detecting the onset of hyper-reflexive bladder contractions from the electrical activity of the pudendal nerve. IEEE Trans. Neural syst. Rehabil. Eng..

[32] Mathews KS (2014). Acute monitoring of genitourinary function using intrafascicular electrodes: selective pudendal nerve activity corresponding to bladder filling, bladder fullness, and genital stimulation. Urology..

[33] Jezernic S, Wen JG, Rijkhoff NJ, Djurhuus JC, Sinkjaer T (2000). Analysis of bladder related nerve cuff electrode recordings from preganglionic pelvic nerve and sacral roots in pigs. J. Urol..

[34] Kurstjens GAM, Borau A, Rodriguez A, Rijkhoff NJM, Sinkjaer T (2005). Intraoperative recording of electroneurographic signals from cuff electrodes on extradural sacral roots in spinal cord injured patients. J. Urol..

[35] Bruns TM, Gaunt RA, Weber DJ (2011). Multielectrode array recordings of bladder and perineal primary afferent activity from the sacral dorsal root ganglia. J. Neural Eng..

[36] Mendez A, Sawan M, Minagawa T, Wyndaele JJ (2013). Estimation of bladder volume from afferent neural activity. IEEE Trans. Neural syst. Rehabil. Eng..

[37] Weber DJ, Stein RB, Everaert DG, Prochazka A (2007). Limb-state feedback from ensembles of simultaneously recorded dorsal root ganglion neurons. J. Neural Eng..

[38] Stein RB (2004). Coding of position by simultaneously recorded sensory neurones in the cat dorsal root ganglion. J. Physipl..

[39] Wagenaar JB, Ventura V, Weber DJ (2011). State-space decoding of primary afferent neuron firing rates. J. Neural Eng..

[40] Rigosa J, Weber DJ, Prochazka A, Stein RB, Micera S (2011). Neuro-fuzzy decoding of sensory information from ensembles of simultaneously recorded dorsal root ganglion neurons for functional electrical stimulation applications. J. Neural Eng..

[41] Weber DJ, Stein RB, Everaert DG, Prochazka A (2006). Decoding sensory feedback from firing rates of afferent ensembles recorded in cat dorsal root ganglia in normal locomotion. IEEE Trans. Neural syst. Rehabil. Eng..

[42] Umeda T (2014). Decoding of the spike timing of primary afferents during voluntary arm movements in monkeys. Front. Neurosci..

[43] Bruns TM, Wagenaar JB, Bauman MJ, Gaunt RA, Weber DJ (2013). Real-time control of hind limb functional electrical stimulation using feedback from dorsal root ganglia recordings. J. Neural Eng..

[44] Holinski BJ, Everaert DG, Mushahwar VK, Stein RB (2013). Real-time control of walking using recordings from dorsal root ganglia. J. Neural Eng..

[45] Han S, Chu JU, Kim H, Park JW, Youn I (2017). Multiunit activity-based real-time limb-state estimation from dorsal root ganglion recordings. Sci. Rep-UK..

[46] Khurram A (2017). Chronic monitoring of lower urinary tract activity via a sacral dorsal root ganglia interface. J. Neural Eng..

[47] Park JH (2013). Detecting bladder fullness through the ensemble activity patterns of the spinal cord unit population in a somatovisceral convergence environment. J. Neural Eng..

[48] Hochreiter S, Schmidhuber J (1997). Long short-term memory. Neural Comput..

[49] Chen Y, Lin Z, Zhao X, Wang G, Gu Y (2014). Deep learning-based classification of hyperspectral data. IEEE J. Sel. Topics Appl. Earth Observ. Remote Sens..

[50] Greff K, Srivastava RK, Koutník J, Steunebrink BR, Schmidhuber J (2017). LSTM: A search space odyssey. IEEE Trans. Neural Netw. Learn. Syst..

[51] Patterson, J. & Gibson, A. Deep Learning: A Practitioner’s Approach, First edition (2017).

[52] Tsoi AC, Back AD (1994). Locally recurrent globally feedforward networks: a critical review of architectures. IEEE Trans. Neural Netw..

[53] Hochreiter, S., Bengio, Y., Frasconi, P. & Schmidhuber, J. Gradient Flow in Recurrent Nets: the Difficulty of Learning Long-Term Dependencies (2001).

[54] Darbellay GA, Vajda I (1999). Estimation of the information by an adaptive partitioning of the observation space. IEEE Trans. Inf. Theory..

[55] Toossi, A., Everaert, D. G., Azar, A., Dennison, C. R. & Mushahwar, V. K. Mechanically stable intraspinal microstimulation implants for human translation. *ANN. Biomed. Eng*. 1;45(3):681–94, 10.1007/s10439-016-1709-0 (2017). 10.1007/s10439-016-1709-0 27562143

[56] Debnath S, Bauman MJ, Fisher LE, Weber DJ, Gaunt RA (2014). Microelectrode array recordings from the ventral roots in chronically implanted cats. Front. Neurol. Neurosci..

[57] Ersen A, Elkabes S, Freedman DS, Sahin M (2015). Chronic tissue response to untethered microelectrode implants in the rat brain and spinal cord. J. Neural Eng..

[58] Kim T, Branner A, Gulati T, Giszter SF (2013). Braided multi-electrode probes: mechanical compliance characteristics and recordings from spinal cords. J. Neural Eng..

[59] Kipke DR, Vetter RJ, Williams JC, Hetke JF (2003). Silicon-substrate intracortical microelectrode arrays for long-term recording of neuronal spike activity in cerebral cortex. IEEE Trans. Neural syst. Rehabil. Eng..

[60] Moxon KA, Leiser SC, Gerhardt GA, Barbee KA, Chapin JK (2004). Ceramic-based multisite electrode arrays for chronic single-neuron recording. IEEE Trans. Biomed. Eng..

[61] Rousche PJ, Normann RA (1998). Chronic recording capability of the Utah Intracortical Electrode Array in cat sensory cortex. J. Neurosci. Methods..

[62] Zhong Y, Bellamkonda RV (2007). Dexamethasone-coated neural probes elicit attenuated inflammatory response and neuronal loss compared to uncoated neural probes. Brain Res..

